# Atypical presentation of *Neisseria meningitidis* serogroup W disease is associated with the introduction of the 2013 strain

**DOI:** 10.1017/S0950268821001035

**Published:** 2021-04-29

**Authors:** Olof Säll, Bianca Stenmark, Susanne Jacobsson, Lorraine Eriksson, Sara Thulin Hedberg, Olof Hertting, Hans Fredlund, Martin Sundqvist, Paula Mölling

**Affiliations:** 1 Department of Infectious Diseases, Faculty of Medicine and Health, Örebro University, SE 70182 Örebro, Sweden; 2 Department of Laboratory Medicine, Clinical Microbiology, Faculty of Medicine and Health, Örebro University, SE 70182 Örebro, Sweden; 3 Paediatric Infectious Diseases Unit, Department of Paediatrics, Astrid Lindgren Children's Hospital, Karolinska University Hospital, SE 17176 Stockholm, Sweden

**Keywords:** Invasive meningococcal disease, meningococcal disease, *Neisseria meningitidis*, serogroup W, Sweden

## Abstract

Since 2015, the incidence of invasive meningococcal disease (IMD) caused by serogroup W (MenW) has increased in Sweden, due to the introduction of the 2013 strain belonging to clonal complex 11. The aim of this study was to describe the clinical presentation of MenW infections, in particular the 2013 strain, including genetic associations. Medical records of confirmed MenW IMD cases in Sweden during the years 1995–2019 (*n* = 113) were retrospectively reviewed and the clinical data analysed according to strain. Of all MenW patients, bacteraemia without the focus of infection was seen in 44%, bacteraemic pneumonia in 26%, meningitis in 13% and epiglottitis in 8%, gastrointestinal symptoms in 48% and 4% presented with petechiae. Phylogenetic analysis was used for possible links between genetic relationship and clinical picture. The 2013 strain infections, particularly in one cluster, were associated with more severe disease compared with other MenW infections. The patients with 2013 strain infections (*n* = 68) were older (52 years *vs*. 25 years for other strains), presented more often with diarrhoea as an atypical presentation (*P* = 0.045) and were more frequently admitted for intensive care (*P* = 0.032). There is a risk that the atypical clinical presentation of MenW infections, with predominantly gastrointestinal or respiratory symptoms rather than neck stiffness or petechiae, may lead to delay in life-saving treatment.

## Introduction

*Neisseria meningitidis* is a strictly human pathogen and the causative agent of invasive meningococcal disease (IMD), which classically presents as septicaemia with or without meningitis. The mortality of IMD is high, even if antibiotic treatment is started early because the virulence of the bacteria initiates a massive immune reaction within the host [1, 2].

Based on the capsular polysaccharide composition, 13 serogroups of *N. meningitidis* have been defined, and of these, six serogroups (A, B, C, W, Y and X) cause the majority of diseases [2]. The different serogroups have been associated with varying epidemiology; for example, serogroup A (MenA) and serogroup C (MenC) predominately cause meningitis among children and adolescents, while serogroup Y (MenY) affects older people and may cause lower respiratory tract infections [3–5].

In Europe, serogroup B (MenB) and MenC have been the most common serogroups causing IMD in recent decades. However, since 2012, serogroup W (MenW) infections have increased in several European countries including Sweden, the UK, the Netherlands, France and Switzerland [6–8]. One reason for the increased incidence of MenW infections is the introduction and spread of a particular strain, the ‘2013 strain’ (which is also referred to as the ‘novel UK strain’ or the ‘UK 2013 strain’) named after the year of its first appearance in the UK [9, 10]. This strain belongs to clonal complex 11 (cc11), is part of the MenW cc11 South American substrain and features the fine type PorA subtype P1.5,2,36-2 and FetA F1-1 [10]. Since 2015, the 2013 strain is the most common individual strain causing IMD in Sweden.

Previous studies have described atypical clinical pictures of MenW infections including gastrointestinal symptoms at presentation [11–14]. In a French study of MenW infections, a subgroup of 2013 strain infections was associated with a high case fatality rate and rarely affected children [8]. However, there is still limited knowledge of whether the clinical presentation differs between the 2013 strain and other MenW strains.

The aim of this study was to investigate the clinical presentation of MenW infections in Sweden during the years 1995–2019, with a special focus on the 2013 strain and its phylogenetics.

## Methods

### Collection of clinical data

Invasive meningococcal infections, defined according to the European Union case definition [15], are mandatorily reported in Sweden. All reported cases of MenW IMD in Sweden from 1995 to 2019 were eligible for inclusion in this retrospective observational study. A uniform questionnaire was used to collect the clinical data from medical records. The medical records were reviewed by the County Medical Officers for Communicable Disease Control and Prevention across Sweden, or by a medically trained person delegated by the County Medical Officer. The authors then compiled data from the completed questionnaires for analysis.

The questionnaire included patient characteristics (e.g. age, gender, household conditions and smoking) to study risk factors for MenW IMD [1, 16, 17]. Symptoms from the onset of acute disease and in-hospital findings including laboratory results were recorded to study the clinical presentations. The questionnaire also covered the sites of infection as well as different outcomes, including mortality and need for intensive care and assisted ventilation. All-cause mortality within 30 days of admission was collected at the time of investigation and matched with the Swedish death registry [18].

The assessments of the clinical presentations were based on a retrospective review of the medical records. The diagnosis of meningitis was based on positive cerebrospinal fluid culture or polymerase chain reaction (PCR), with or without positive blood cultures. In cases where lumbar puncture was not performed, the diagnosis was deemed as meningitis if the clinical picture suggested meningitis in combination with bacteraemia. The diagnosis of bacteraemic pneumonia was based on clinical symptoms and radiological findings suggesting pneumonia in combination with blood cultures positive for MenW. Upper respiratory tract infections were recorded if they were considered clinically relevant by the clinician and were combined with MenW bacteraemia. Arthritis was assessed if either culture or PCR on joint fluid was positive for MenW. Bacteraemia without apparent focus was recorded when blood culture was positive for MenW but no localised clinical manifestation was found. Cultures from the airways, including bronchoalveolar culture, were not included in this study. In addition, it was not possible to classify sepsis severity in this retrospective study spanning a timeline with varying sepsis definitions.

### Bacterial isolates and genomic analysis

In Sweden, all clinical IMD isolates are routinely sent to the National Reference Laboratory for *N. meningitidis* at Örebro University Hospital, Örebro, Sweden, for susceptibility testing and subtyping using whole-genome sequencing on the Illumina platform, as previously described [9]. These genomes are continuously deposited in the *Neisseria* spp. pubMLST database[19]. The MenW genomes included in this study from the period 1995–2017 have previously been described by Eriksson *et al*. [9] and the isolates from 2018 to 2019 were sequenced using the same methods as part of the routine diagnostics. PubMLST IDs of all isolates are shown in Supplementary material S1.

Illumina reads from the MenW isolates were mapped onto a PacBio sequenced MenW genome deposited in the pubMLST database (pubMLST ID 82050) using CLC Genomics Workbench v. 20.0 (Qiagen, Venlo, The Netherlands). Single nucleotide polymorphism (SNP) trees were created using default parameters and the Neighbour Joining algorithm in CLC Genomics Workbench.

### Data analysis and statistics

To investigate possible differences in the clinical picture that might be explained by different genotypes, the patient cohort infected by the 2013 strain was compared with the group of MenW patients with non-2013 strain infections. In addition, the cohort of cc11 infections was compared with non-cc11 infections; data are available in Supplementary material S2. Also, 2013 strain infections were compared with other cc11 isolates (i.e. original UK strain isolates); data are presented in Supplementary material S3. To determine statistical significance between groups, the Pearson *χ*^2^ method was used, or Fisher's exact test if the sample size was small. The Mann–Whitney *U*-test was used for the comparison of non-parametric median values.

Selected outcomes (mortality, need for intensive care or assisted ventilation) were compared with the exposure to various strains (2013 strain or other strains). In this analysis, logistic regression analysis was used with an adjustment on age and sex. In the adjustment analysis for the possible effect of age on outcomes, the patients were categorised into three age categories. Statistical analyses were performed using SPSS statistical software (IBM SPSS statistics version 25, IBM Corporation, Armonk, NY, USA).

### Ethics

The study was approved by the Regional Ethical Review Board in Uppsala (reference number 2018/139) and an amendment by the Swedish Ethical Review Authority (reference number 2019-05697).

## Results

**Fig. 1.**
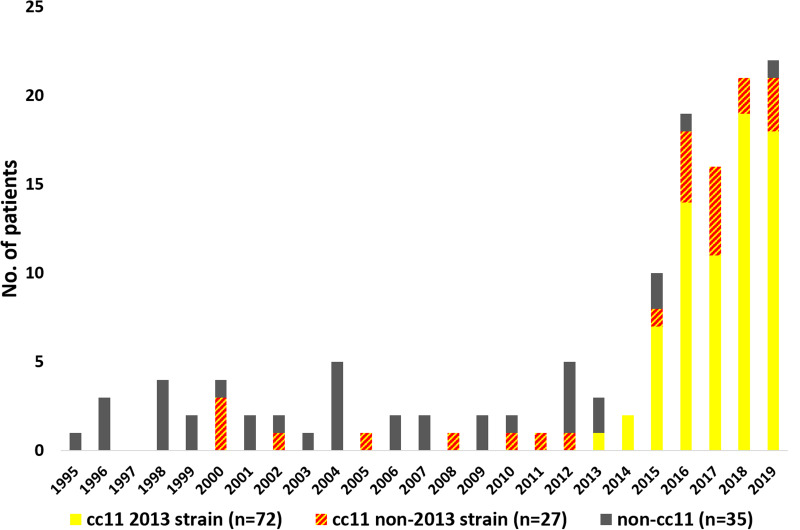
Number of reported *N. meningitidis* MenW cases per year in Sweden 1995–2019 with the distribution of strains, *n* = 134.

It was possible to access medical records for 113/134 (84%) patients with MenW IMD in Sweden 1995–2019 (Fig. 1). The majority of the missing records were from two counties/regions and the inaccessibility was due to a change of record systems. As the missing cases were from earlier years of the study, 17/21 (81%) were caused by non-2013 strain MenW. The basic characteristics of the missing patients were similar to those of the included patients (Supplementary material S4). Among the medical records accessed, not all individual parameters could be found, explaining different denominators.

During the study period 1995–2019, the total IMD incidence in Sweden varied between 0.4 and 1.1 cases per 100 000 inhabitants per year. All MenW cases were sporadic except for two minor outbreaks with epidemiologically linked cases; two cases in relation to an international scout jamboree in 2015 (cc11, 2013 strain [10] and three cases within a limited community group (cc11, non-2013 strain). The cases were spread over the years without any seasonal peak (Supplementary Table S1).

**Fig. 2.**
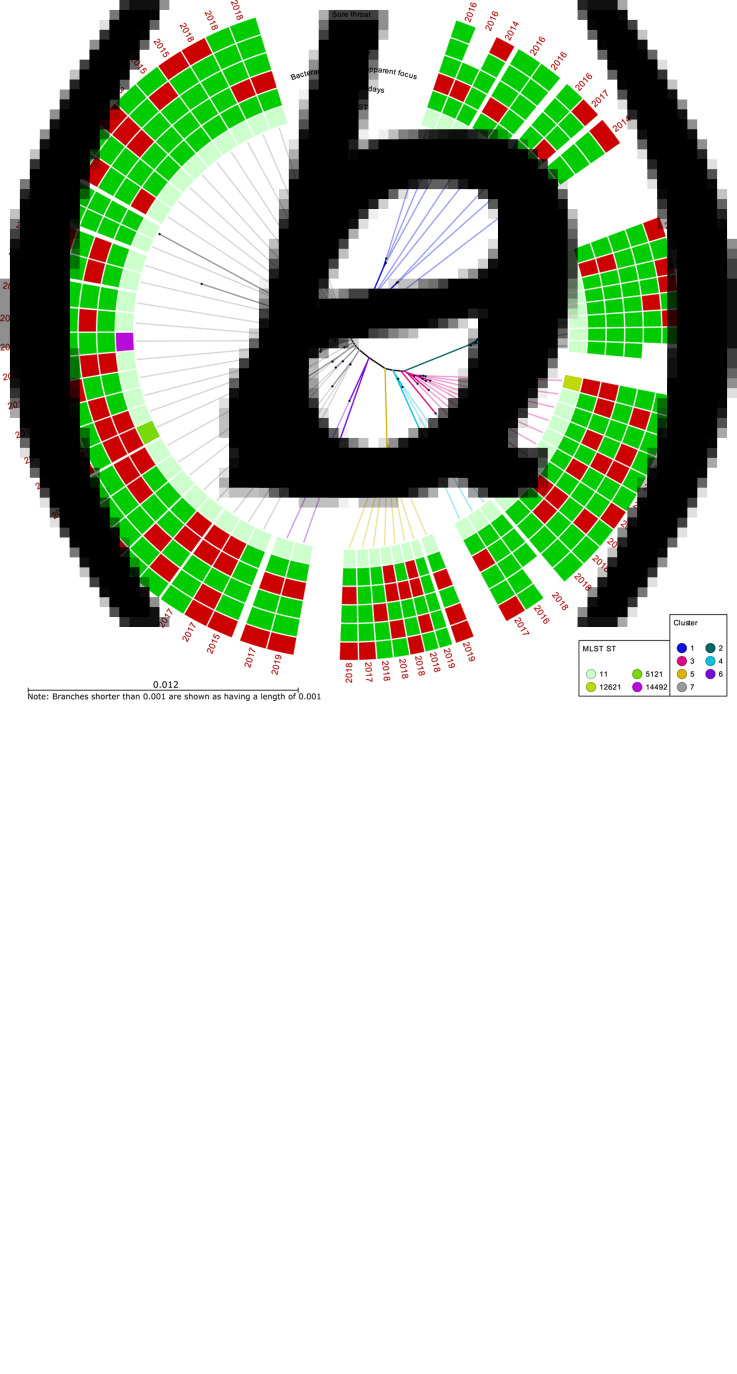
Neighbour-joining phylogenetic SNP trees of (A) all MenW isolates (*n* = 113) in a circular phylogram and (B) 2013 strain isolates exclusively (*n* = 68) in radial view. Strain designation according to Lucidarme *et al*. [10]. Multilocus sequencing typing clonal complexes (cc) or sequence types, as well as patient outcome and symptoms, are displayed in tiles. Isolates from patients with the outcome or symptom are shown in red and those without are shown in green. The year of isolation is shown for each node.

**Fig. 3.**
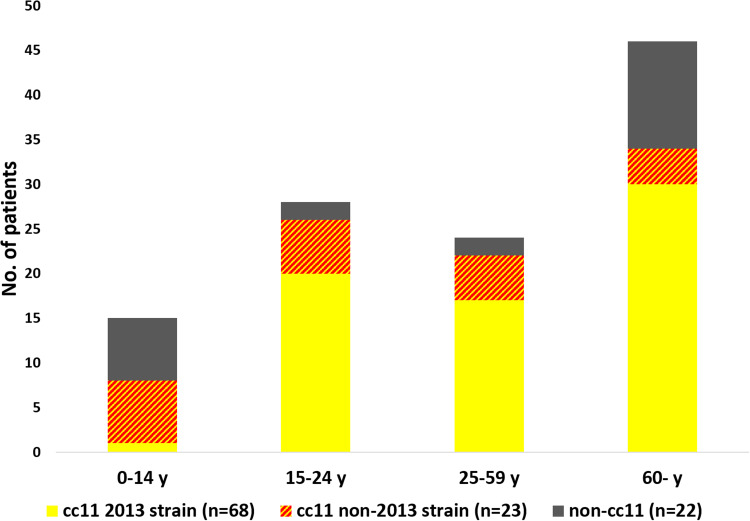
Number of patients per age group with invasive meningococcal MenW disease in Sweden 1995–2019 where medical records were obtained (*n* = 113), including distribution of strains within the age groups.

The majority of the included MenW isolates belonged to cc11 (91/113, 81%), of which 68 isolates belonged to the 2013 strain (Fig. 2A). Of all included patients, the median age was 48 years, with a higher median age of 52 years for patients with 2013 strain infections, compared with 25 years for other strains (*P* = 0.032) (Table 1, Fig. 3). In addition, children under 18 years were less often infected by the 2013 strain; 8/68 (12%) of the 2013 strain infections affected children under 18 years compared with 17/45 (38%) for other isolates (*P* = 0.001). Except for age, there were no differences between the patient cohorts (2013 strain *vs.* non-2013 strain). Of all MenW patients, 16/113 (14%) were immunocompromised and 9/81 (11%) were smokers.

**Table 1.** Characteristics and clinical parameters of patients (*n* = 113) with meningococcal disease caused by the 2013 strain compared with other MenW strains

	Total (*n* = 113)	2013 strain (*n* = 68)	Non-2013 strain (*n* = 45)	*P* value
*Baseline characteristics*				
Age: median (25th–75th percentile), range, years	48 (19–73) 0–95	52 (20–73) 12–95	25 (4–74) 0–94	**0**.**032**
Female gender	65/113 (58%)	40/68 (59%)	25/45 (56%)	0.731
Living alone	30/105 (29%)	15/60 (25%)	15/45 (33%)	0.350
Smoker^a^	9/81 (11%)	4/48 (8%)	5/33 (15%)	0.337
Immunocompromised state^b^	16/113 (14%)	11/68 (16%)	5/45 (11%)	0.450
*Symptoms and clinical findings*				
Diarrhoea	20/110 (18%)	16/65 (25%)	4/45 (9%)	**0**.**045**
Vomiting	40/110 (36%)	24/65 (37%)	16/45 (36%)	0.883
Diarrhoea and/or vomiting	48/110 (44%)	29/65 (45%)	19/45 (42%)	0.803
Headache	23/110 (21%)	17/65 (26%)	6/45 (13%)	0.104
Sore throat	38/110 (35%)	28/65 (43%)	10/45 (22%)	**0**.**024**
Cough	26/110 (24%)	18/65 (28%)	8/45 (18%)	0.229
Decreased level of consciousness	23/112 (21%)	15/65 (23%)	10/43 (23%)	0.983
Petechiae	4/100 (4%)	2/60 (3%)	2/40 (5%)	1.000
*Laboratory results*^c^				
Platelet count <150 × 10^9^/l	44/105 (42%)	33/64 (52%)	11/41 (27%)	**0**.**012**
Lactate >4 mmol/l	34/74 (46%)	28/53 (53%)	6/21 (29%)	0.059
*Time from onset of symptoms to arrival at hospital*				
Median (25th–75th percentile), days	1 (0–2)	1 (0–2)	1 (0–2)	0.569
*Time from arrival at hospital to antibiotic treatment*				
Median (25th–75th percentile), hours	1.5 (1.0–4.0)	1.0 (0.5–3.0)	2.5 (1.0–4.0)	**0**.**040**
*Sites of infection*^d^				
Meningitis	15/113 (13%)	5/68 (7%)	10/45 (22%)	**0**.**023**
Pneumonia	26/113 (23%)	15/68 (22%)	11/45(24%)	0.768
Arthritis	6/113 (5%)	3/68 (4%)	3/45 (7%)	0.681
Throat infection	18/113 (16%)	13/68 (19%)	5/45 (11%)	0.255
Epiglottitis	9/113 (8%)	6/68 (9%)	3/45 (7%)	1.000
Bacteraemia without apparent focus	50/113 (44%)	33/68 (49%)	17/45 (38%)	0.260

Differences between groups analysed using the *χ*^2^ test or Fisher's exact test. For comparison of non-parametric median values, the Mann–Whitney *U*-test was used. P < 0.05 was considered statistically significant and is marked in bold.

a Active smoker or passively exposed to cigarette smoke.

b Due to diabetes (*n* = 8), malignancy (*n* = 3), splenectomised state (*n* = 2), immunosuppressive treatment (immunomodulating drugs, cytostatic drugs or corticosteroids equivalent to ⩾10 mg prednisolone daily) (*n* = 2) or alcohol abuse (*n* = 1). None had verified complement deficiency.

c Results of the first test at hospital.

d The assessments of the sites of infection were based on the retrospective review of the medical records. Except for bacteraemia without apparent focus, more than one site of infection is possible.

### Clinical presentation and disease severity

The symptoms of MenW infections are presented in Table 1 and include gastrointestinal symptoms (48/110, 44%), sore throat (38/110, 35%) and petechiae (4/100, 4%). The most common final diagnoses were bacteraemia with no apparent site of infection (50/113, 44%) and bacteraemic pneumonia (26/113, 23%). Meningitis was seen in 15/113 (13%) of the patients and was less common among 2013 strain patients (5/68, 7%) compared with other strains (10/45, 22%) (*P* = 0.023). Of the patients, 18/113 (16%) had severe throat infection including 9/113 (7%) with epiglottitis (Table 1). Except for epiglottitis, other reported throat infections included laryngitis (*n* = 3), tonsillitis (*n* = 3) and peritonsillitis (*n* = 1). Reported sore throat with or without evident throat infection was more common in the 2013 strain cohort (28/65, 43%) compared with other isolates (10/45, 22%) (*P* = 0.024); however, clinically diagnosed throat infections were not significantly higher among the 2013 strain infections (13/68, 19%) compared with infections caused by other strains (5/45, 11%) (*P* = 0.255) (Table 1). Meningitis was more common in the lower age group with a median age of 11 years compared with 54 years for patients without meningitis (*P* < 0.001), while epiglottitis patients were older, with a median age of 73 years compared with 37 years for patients without epiglottitis (*P* = 0.007) (Table 2). Among patients with bacteraemic pneumonia, 2/26 (8%) required assisted ventilation (Supplementary material S5).

**Table 2.** Age in relation to site of infection for all meningococcal MenW infections and 2013 strain infections, respectively

	Age: median (25th–75th percentile), range, years		
	Infection focus present	Infection focus absent	*P* value
Total (*n* = 113)			
Bacteraemia without apparent focus (*n* = 50)	44 (20–74), 0–86	48 (17–73), 0–95	0.707
Meningitis^a^ (*n* = 15)	11 (1–17), 0–34	54 (21–74), 0–95	**<0**.**001**
Pneumonia^b^ (*n* = 26)	64 (20–80), 2–94	39 (18–72), 0–95	0.085
Epiglottitis^b^ (*n* = 9)	73 (62–80), 60–84	37 (18–72), 0–95	**0**.**007**
Arthritis^c^ (*n* = 6)	34 (3–60), 1–94	49 (19–73), 0–95	0.423
2013 strain (*n* = 68)			
Bacteraemia without apparent focus (*n* = 33)	49 (21–72), 16–84	60 (20–73), 12–95	0.335
Meningitis^a^ (*n* = 5)	17 (14–33), 12–34	57 (21–73), 16–95	**0**.**009**
Pneumonia^b^ (*n* = 15)	69 (20–83), 15–94	49 (21–72), 12–95	0.122
Epiglottitis^b^ (*n* = 6)	71 (62–78), 60–83	50 (20–72), 12–95	**0**.**042**
Arthritis^c^ (*n* = 3)	48 (20–48), 20–49	56 (21–73), 12–95	0.403

The Mann–Whitney *U*-test was used for comparison between patients with the site of infection *vs*. patients without the site of infection respectively. *P* < 0.05 was considered statistically significant and is marked in bold.

a Meningitis was assessed if MenW was detected in cerebrospinal fluid or clinical picture of meningitis in combination with MenW bacteraemia.

b Pneumonia and epiglottitis were both combined with bacteraemia.

c Arthritis was assessed if MenW was detected in joint fluid by culture or PCR.

Of all patients, 16/113 (14%) died within 30 days of admission, with 9/16 (56%) of these dying within 24 h of admission (Table 3). The highest mortality was seen in the group of patients with bacteraemia without apparent focus of infection (13/50, 26%) (Supplementary material S5).

**Table 3.** Meningococcal disease severity for 2013 strain compared with infections caused by other MenW strains

	*n* (%)	OR (95% CI), unadjusted	*P* value	OR (95% CI), adjusted by age and sex	*P* value
Mortality <30 days					
Total	16/113 (14%)				
2013 strain	13/68 (19%)	3.3 (0.9–12.4)	0.075	3.6 (0.9–13.7)	0.066
Non-2013 strain	3/45 (7%)	Ref		Ref	
Mortality <24 h					
Total	9/113 (8%)				
2013 strain	8/68 (12%)	5.9 (0.7–48.6)	0.101	6.0 (0.7–49.9)	0.100
Non-2013 strain	1/45 (2%)	Ref			
Intensive care					
Total	60/110 (55%)				
2013 strain	41/66 (62%)	2.2 (1.0–4.7)	0.052	2.4 (1.1–5.4)	**0**.**032**
Non-2013 strain	19/44 (43%)	Ref		Ref	
Assisted ventilation					
Total	25/106 (24%)				
2013 strain	19/63 (30%)	2.7 (1.0–7.4)	0.059	2.7 (1.0–7.4)	0.061
Non-2013 strain	6/44 (14%)	Ref		Ref	

Adjustment for age and sex was performed using logistic regression. *P* < 0.05 was considered statistically significant and is marked in bold.

A non-significant higher 30-day mortality was observed in the 2013 strain cohort (OR 3.6, 95% CI 0.9–13.7) compared with other isolates (*P* = 0.075). Although the 2013 strain patients were older, age and sex-adjusted analysis showed that the higher mortality in the 2013 strain cohort was not due to age or sex. Among all MenW patients, intensive care admission was required for 60/110 (55%) patients. Adjustment for age and sex showed that the need for intensive care was significantly greater among 2013 strain patients (*P* = 0.032). Assisted ventilation was required for 25/106 (24%) patients, with a non-significant trend to be more common among 2013 strain infections compared with other strains (OR 2.7 (95% CI 1.0–7.4), *P* = 0.061) (Table 3).

Regarding biomarkers associated with disease severity, low platelet count (<150 × 10^9^/l) was more common among the 2013 strain cohort (33/64, 52%) compared with other patients (11/41, 27%) (*P* = 0.012) (Table 1). Low platelet count, low white blood cell count and elevated lactate levels in the blood at admission were all independently associated with higher mortality (Supplementary material S6).

In addition to comparing the 2013 strain infections with other MenW isolates, cc11 infections were compared with non-cc11 infections. In brief, 30-day mortality was seen in 15/91 (17%) of the cc11 patients compared with 1/22 (5%) for other patients (*P* = 0.190) and need for intensive care was seen in 54/88 (61%) of the cc11 patients compared with 6/22 (27%) for non-cc11 infections (*P* = 0.004). Also, meningitis was seen less often among cc11 patients compared with non-cc11 patients (Supplementary material S2). Likewise, the 2013 strain infections were compared with all other isolates within cc11 (Suppl. S3). This showed similar results as the analysis of 2013 strain infections compared with all other MenW isolates, however with a smaller control group limiting statistical power.

### Phylogenetic analysis

The genetic relationship between the MenW isolates is presented in Figure 2. The isolates within cc11 were very similar, separated by ~0–4000 SNPs. The isolates in the 2013 strain differ from the isolates of other ccs by ~15 000– 17 000 SNPs.

Clusters consisting of closely related isolates within the 2013 strain were identified, with only small genetic differences between the individual clusters – the isolates within the 2013 strain did not differ by more than 1130 SNPs. Clusters were analysed for possible associations with clinical data. One cluster that consisted of 29 isolates (cluster 7, Fig. 2B) was associated with higher mortality (*P* = 0.016) and a greater need for intensive care treatment (*P* = 0.038), compared with other MenW isolates, and cluster 1 (*n* = 8) was associated with fewer gastrointestinal symptoms (*P* = 0.018) (Fig. 2B, Table 4).

**Table 4.** Associations between clusters within *N. meningitidis* MenW 2013 strain and clinical data

	The cluster respectively	All other MenW	*P* value
Cluster 1 (*n* = 8)			
Vomiting	0/7 (0%)	40/103 (39%)	**0**.**047**
Diarrhoea and/or vomiting	0/7 (0%)	48/103 (47%)	**0**.**018**
Lactate >3 mmol/l	1/8 (13%)	40/66 (61%)	**0**.**019**
Cluster 2 (*n* = 8)			
Cluster 3 (*n* = 10)			
Cluster 4 (*n* = 3)			
Cluster 5 (*n* = 8)			
Assisted ventilation	5/8 (63%)	25/106 (24%)	**0**.**017**
Cluster 6 (*n* = 2)			
Cluster 7 (*n* = 29)			
Mortality <30 days	8/29 (28%)	8/84 (10%)	**0**.**016**
Mortality <24 h	5/29 (17%)	4/84 (5%)	**0**.**047**
Intensive care	20/28 (71%)	40/82 (49%)	**0**.**038**
Epiglottitis	5/29 (17%)	4/84 (5%)	**0**.**047**

Each cluster has been compared with all other MenW isolates using the *χ*^2^ test, or Fisher's exact test in the case of small sample numbers. Only statistically significant associations are presented.

## Discussion

In this study, we analysed the clinical presentation of 2013 strain infections compared with other MenW isolates, and we interpret that the 2013 strain may cause more severe disease. This might indicate that the spread and increase of the 2013 strain is a key reason for the higher mortality associated with MenW in recent years.

High mortality rates for all MenW infections (14%) were found, especially in the 2013 strain cohort (19%). In addition, the need for intensive care was high, both for all patients (55%) and for the 2013 strain (62%) cohort, which is more than previously described for MenW infections [13, 14]. Our results are consistent with the report from Hong *et al*. that showed MenW cc11 infections to be potentially more aggressive than non-cc11 MenW isolates [8]. Here, we found that not only MenW cc11 isolates, but also the 2013 strain, caused more severe disease compared with other MenW isolates.

Another important finding in this study was the atypical presentations of MenW infections. Gastrointestinal symptoms were seen in almost every other patient (44%), and diarrhoea was a more common initial presentation of 2013 strain IMD compared with infections caused by other MenW strains. Vomiting and/or diarrhoea have been described as dominating presenting symptoms in MenW IMD, potentially leading to delays in management, as IMD was not initially suspected [13, 14, 20, 21]. Meningitis, which is regarded as a typical presentation of IMD, was seen in only 13%, which is relatively low compared with other studies on MenW patients – 23% in a recent Dutch study [14] and 28% among MenW patients in England and Wales [13]. Further, meningitis was significantly less frequent in the 2013 strain compared with other isolates. Instead, atypical infection sites were common, such as bacteraemic pneumonia (23%) and throat infections (16%) including epiglottitis (8%), which is in line with previous reports [13]. Among patients with bacteraemic pneumonia, the need for assisted ventilation was low, suggesting MenW caused a mild form of pneumonia. It is worth noting that only 4% of the patients in this study presented with petechiae, regarded as a typical sign of IMD. The absence of petechiae may lead to potentially delayed recognition of IMD and therefore delay in treatment. Although only a limited number of patients presented with petechiae, 42% of all MenW patients had a low platelet count, which can be a sign of severe disease. Petechiae have been more commonly reported among MenB infections and less commonly among serogroups Y and W [4, 14]. A recent study from the UK, where meningococcal MenB and MenC vaccination is implemented, found IMD now to be a rare cause of petechiae in children presenting with fever [22].

A phylogenetic tree was created to study links between isolate relationship and clinical picture. We found one cluster to be associated with more severe disease, while another cluster was negatively associated with gastrointestinal symptoms. These findings indicate that genetic differences within lineages may explain the variety in clinical expressions, which calls for further studies on whether this is due to certain genes or SNPs.

In Sweden, the epidemiology of IMD has changed considerably in the last decade. Before 2009, MenB and MenC dominated and affected mostly younger children and adolescents. From 2009 to 2014, there was an increase in MenY infections due to the introduction of a new strain to Sweden. Since 2015, after the introduction of the MenW 2013 strain, most IMD in Sweden has been caused by MenY or MenW, and IMD now affects all age groups. When comparing these two serogroups in Sweden, MenY patients are older than MenW patients (median age 62 years *vs.* 48 years), with a higher proportion of age-related immunocompromised status (25% *vs.* 14%) [4]. In the present study, children under 18 years were less often affected by the 2013 strain and only one child was under 14 years. It is not known why this strain rarely infects children. The higher mortality rates in Sweden for MenW infections (14%) compared with MenY infections (9%) [4] are consistent with studies on a mouse model expressing human transferrin infected with these isolates, in which MenW isolates induced higher bacteraemia levels and proinflammatory activity as well as a higher degree of apoptosis [23].

Disease severity in meningococcal infections is determined by host defence and susceptibility, bacterial virulence and environmental factors. Environmental factors could include seasonality, but was not observed in this study. Host factors include impaired immune response; however, in this study, there were no differences in the immunocompromised state between the 2013 strain cohort and other strains. Importantly, the 2013 strain affected older persons, although the higher mortality rate associated with this strain was not due to age differences. The introduction of a new meningococcal strain into a population may be enhanced if the population lacks immunity against that strain. Carriage of *N. meningitidis* is common and provides immunity against IMD, at least against strains that resemble the carriage strains [24]. If the 2013 strain is new for the Swedish population, it could lead to a high attack rate for the exposed individuals. Genome-wide association studies could be one useful tool to increase the understanding of whether certain genetic factors contribute to carriage or disease.

One strength of this national study was that a majority of patients with MenW IMD in Sweden during the study period was included, rather than only a selected sample of cases. Although not all medical records could be retrieved, we assess that excluded and included patients did not differ in their socio-demographic characteristics. The retrospective study design is a limitation. However, it was not possible to perform a prospective study design since Sweden is a low-endemic country for IMD. Another limitation in this study is the small number of isolates in each cluster, reducing statistical power.

In conclusion, this study has shown a change in the epidemiology and clinical presentations of MenW infections in Sweden, driven by the emergence of the 2013 strain. This strain is associated with more severe disease, affects older patients and manifests usually as pneumonia, throat infection or bacteraemia without apparent focus, rather than meningitis. We identified one dominating cluster within the 2013 strain causing more severe disease, a finding that calls for further studies. It is worth noting that this study showed a large degree of atypical clinical presentations of MenW infections, with gastrointestinal symptoms common and petechiae rare. These atypical presentations may lead to delayed detection and treatment, as well as delayed administration of chemoprophylaxis to close contacts.

## Supplementary material

For supplementary material accompanying this paper visit http://dx.doi.org/10.1017/S0950268821001035. click here to view supplementary material

## Data

Genome data used in this study are publically available in the *Neisseria* spp. pubMLST database (https://pubmlst.org/organisms/neisseria-spp). For access to other data used in the current study, the readers are kindly asked to contact the corresponding author.
