# Supplementary material for: Atypical presentation of Neisseria meningitidis serogroup W disease is associated with the introduction of the 2013 strain
Source: Epidemiol Infect. 2021 Apr 29;149:e126. doi: 10.1017/S0950268821001035 (PMC8161285; doi:10.1017/S0950268821001035)
Supplement: Supplementary file 1 [file S0950268821001035sup001.docx]

Epidemiology and Infection

Atypical presentation of *Neisseria meningitidis* serogroup W disease is associated with the introduction of the 2013 strain*.*

Olof Säll, Bianca Stenmark, Susanne Jacobsson, Lorraine Eriksson, Sara Thulin Hedberg, Olof Hertting, Hans Fredlund, Martin Sundqvist, Paula Mölling

Supplementary Table S1. PubMLST IDs of all *Neisseria meningitidis* serogroup W genomes included in the study (n=113) with the corresponding strain, clonal complex, sequence type and month of sampling.

| **pubMLST ID** | **Strain** | **MLST cc** | **MLST ST** | **Month of sampling** |
| --- | --- | --- | --- | --- |
| 45025 | 2013 strain | ST-11 complex | 11 | July |
| 47178 | other | ST-11 complex | 247 | November |
| 58172 | other | ST-11 complex | 11 | October |
| 38614 | 2013 strain | ST-11 complex | 11 | August |
| 50863 | 2013 strain | ST-11 complex | 11 | October |
| 47197 | other | ST-22 complex | 22 | August |
| 38693 | other | not assigned to any cc | 12019 | June |
| 47208 | other | ST-11 complex | 11 | May |
| 38690 | 2013 strain | ST-11 complex | 11 | March |
| 38692 | 2013 strain | ST-11 complex | 11 | May |
| 50872 | other | ST-11 complex | 9435 | December |
| 42363 | 2013 strain | ST-11 complex | 11 | February |
| 42391 | 2013 strain | ST-11 complex | 11 | November |
| 42420 | 2013 strain | ST-11 complex | 11 | January |
| 50875 | other | ST-11 complex | 11 | December |
| 54485 | 2013 strain | ST-11 complex | 11 | June |
| 58169 | 2013 strain | ST-11 complex | 5121 | November |
| 47174 | other | ST-174 complex | 169 | May |
| 47177 | other | ST-11 complex | 11582 | August |
| 53042 | other | ST-22 complex | 184 | March |
| 47179 | other | ST-60 complex | 1243 | January |
| 92886 | other | ST-11 complex | 11 | April |
| 47199 | other | ST-60 complex | 1243 | November |
| 47204 | other | ST-11 complex | 247 | May |
| 47205 | other | ST-22 complex | 184 | July |
| 47211 | other | ST-60 complex | 10212 | October |
| 92887 | other | ST-22 complex | 184 | February |
| 42336 | other | ST-22 complex | 184 | May |
| 38694 | 2013 strain | ST-11 complex | 11 | June |
| 38602 | 2013 strain | ST-11 complex | 11 | August |
| 41964 | 2013 strain | ST-11 complex | 11 | February |
| 92889 | 2013 strain | ST-11 complex | 11 | March |
| 50865 | 2013 strain | ST-11 complex | 11 | November |
| 50975 | other | ST-11 complex | 11 | September |
| 39602 | 2013 strain | ST-11 complex | 11 | April |
| 58165 | 2013 strain | ST-11 complex | 11 | September |
| 58176 | 2013 strain | ST-11 complex | 11 | December |
| 52796 | other | ST-11 complex | 11 | March |
| 52797 | other | ST-11 complex | 11 | April |
| 95841 | other | ST-11 complex | 11 | May |
| 47200 | other | ST-11 complex | 11 | November |
| 42423 | other | ST-22 complex | 12004 | October |
| 53043 | other | ST-22 complex | 22 | April |
| 58164 | 2013 strain | ST-11 complex | 11 | September |
| 41963 | 2013 strain | ST-11 complex | 11 | February |
| 47175 | other | ST-11 complex | 9600 | January |
| 42425 | other | ST-11 complex | 11 | November |
| 42424 | 2013 strain | ST-11 complex | 11 | January |
| 42436 | 2013 strain | ST-11 complex | 11 | February |
| 54487 | 2013 strain | ST-11 complex | 11 | July |
| 47184 | other | ST-22 complex | 1224 | March |
| 47194 | other | ST-60 complex | 1243 | November |
| 47198 | other | ST-22 complex | 184 | July |
| 41966 | other | ST-11 complex | 11 | February |
| 47202 | other | ST-60 complex | 913 | January |
| 47206 | other | ST-11 complex | 1287 | June |
| 47207 | other | ST-22 complex | 184 | February |
| 92888 | 2013 strain | ST-11 complex | 11 | December |
| 38691 | 2013 strain | ST-11 complex | 11 | April |
| 47166 | 2013 strain | ST-11 complex | 12621 | August |
| 46377 | 2013 strain | ST-11 complex | 11 | August |
| 46378 | 2013 strain | ST-11 complex | 11 | August |
| 47167 | 2013 strain | ST-11 complex | 11 | August |
| 47170 | other | ST-22 complex | 22 | September |
| 51910 | 2013 strain | ST-11 complex | 11 | February |
| 47172 | other | ST-174 complex | 185 | April |
| 47173 | other | ST-174 complex | 185 | May |
| 39601 | other | ST-11 complex | 8529 | April |
| 39603 | 2013 strain | ST-11 complex | 11 | May |
| 47201 | other | ST-22 complex | 3661 | January |
| 47210 | other | ST-22 complex | 3661 | September |
| 42786 | 2013 strain | ST-11 complex | 11 | April |
| 54486 | 2013 strain | ST-11 complex | 11 | July |
| 58175 | 2013 strain | ST-11 complex | 11 | December |
| 71427 | 2013 strain | ST-11 complex | 11 | December |
| 84119 | 2013 strain | ST-11 complex | 11 | August |
| 61306 | 2013 strain | ST-11 complex | 11 | May |
| 91879 | 2013 strain | ST-11 complex | 11 | July |
| 91882 | 2013 strain | ST-11 complex | 11 | July |
| 61289 | 2013 strain | ST-11 complex | 11 | February |
| 91888 | 2013 strain | ST-11 complex | 11 | August |
| 84118 | 2013 strain | ST-11 complex | 11 | August |
| 61294 | 2013 strain | ST-11 complex | 11 | March |
| 61299 | 2013 strain | ST-11 complex | 11 | April |
| 85264 | 2013 strain | ST-11 complex | 11 | December |
| 70778 | 2013 strain | ST-11 complex | 11 | September |
| 61292 | 2013 strain | ST-11 complex | 11 | March |
| 61296 | 2013 strain | ST-11 complex | 11 | March |
| 61297 | 2013 strain | ST-11 complex | 11 | March |
| 61300 | 2013 strain | ST-11 complex | 11 | April |
| 84113 | 2013 strain | ST-11 complex | 11 | January |
| 85269 | 2013 strain | ST-11 complex | 11 | January |
| 88980 | 2013 strain | ST-11 complex | 14492 | June |
| 88981 | other | ST-11 complex | 11 | March |
| 91885 | 2013 strain | ST-11 complex | 11 | August |
| 70775 | 2013 strain | ST-11 complex | 11 | August |
| 70785 | 2013 strain | ST-11 complex | 11 | October |
| 85263 | 2013 strain | ST-11 complex | 11 | December |
| 70776 | 2013 strain | ST-11 complex | 11 | August |
| 71922 | 2013 strain | ST-11 complex | 11 | December |
| 84116 | 2013 strain | ST-11 complex | 11 | July |
| 85262 | 2013 strain | ST-11 complex | 11 | November |
| 85271 | other | ST-11 complex | 11 | January |
| 88984 | 2013 strain | ST-11 complex | 11 | May |
| 70774 | 2013 strain | ST-11 complex | 11 | July |
| 83924 | other | ST-11 complex | 11 | September |
| 84123 | other | ST-11 complex | 11 | September |
| 91871 | other | ST-11 complex | 11 | May |
| 84121 | 2013 strain | ST-11 complex | 11 | September |
| 84114 | 2013 strain | ST-11 complex | 11 | July |
| 88974 | 2013 strain | ST-11 complex | 11 | February |
| 91876 | 2013 strain | ST-11 complex | 11 | June |
| 71921 | 2013 strain | ST-11 complex | 11 | December |

MLST = multilocus sequence typing. Cc = clonal complex. ST = sequence type

Supplementary Table S2. Characteristics of patients with meningococcal serogroup W infections (n=113), cc11 infections compared with non-cc11 infections.

|  | **Total**  **(n=113)** | **Cc11 (n=91)** | **Non-cc11 (n=22)** | ***p-*value** |
| --- | --- | --- | --- | --- |
| **Baseline characteristics**  - Age: median (25^th^-75^th^ percentile), range, years  - Female gender  - Living alone  - Smoker^1^  - Immunocompromised state^2^ | 48 (19-73),  0-95  65/113 (58%)  30/105 (29%)  9/81 (11%)  16/113 (14%) | 46 (19-71),  0–95  51/91 (56%)  22/83 (27%)  4/64 (6%)  11/91 (12%) | 64 (2-80),  0–94  14/22 (64%)  8/22 (36%)  5/17 (29%)  5/22 (23%) | 0.655  0.518  0.363  **0.017**  0.199 |
| **Symptoms and clinical findings**  - Diarrhoea  - Vomiting  - Diarrhoea and/or vomiting  - Headache  - Sore throat  - Cough  - Decreased level of consciousness  - Petechiae | 20/110 (18%)  40/110 (36%)  48/110 (44%)  23/110 (21%)  38/110 (35%)  26/110 (24%)  23/112 (21%)  4/100 (4%) | 19/88 (22%)  31/88 (35%)  39/88 (44%)  20/88 (23%)  34/88 (39%)  22/88 (25%)  20/90 (22%)  2/80 (2.5%) | 1/22 (5%)  9/22 (41%)  9/22 (41%)  3/22 (14%)  4/22 (18%)  4/22 (18%)  3/22 (14%)  2/20 (10%) | 0.071  0.620  0.773  0.558  0.083  0.586  0.557  0.126 |
| **Laboratory results^4^**  **WBC count** (x 10^9^/L)  - <4  - 4–12  - >12  **Platelet count** (x 10^9^/L)  - <20  - <50  - <100  - <150  **Lactate** (mmol/L)  - >2  - >3  - >4 | 20/108 (19%)  34/108 (32%)  54/108 (50%)  9/105 (9%)  20/105 (19%)  29/105 (28%)  44/105 (42%)  51/74 (69%)  41/74 (55%)  34/74 (46%) | 18/87 (21%)  25/87 (29%)  44/87 (51%)  9/87 (10%)  19/87 (22%)  27/87 (31%)  42/87 (48%)  49/67 (73%)  39/67 (58%)  34/67 (51%) | 2/21 (10%)  9/21 (43%)  10/21 (48%)  0/18 (0%)  1/18 (6%)  2/18 (11%)  2/18 (11%)  2/7 (29%)  2/7 (29%)  0/7 (0%) | 0.352  0.211  0.808  0.352  0.185  0.145  **0.004**  **0.027**  0.231  **0.013** |
| **Time from onset of symptoms to arrival at hospital**  - Median, (25^th^ -75^th^ percentile), days | 1 (0-2) | 1 (0-2) | 1 (0-2) | 0.779 |
| **Time from arrival at hospital to antibiotic treatment**  - Median, (25^th^ -75^th^ percentile), hours | 1.5 (1-4) | 1.5 (1.0-4.0) | 2.0 (1.0-4.5) | 0.470 |
| **Sites of infection^3^**  - Meningitis  - Pneumonia  - Arthritis  - Throat infection  - Epiglottitis  - Bacteraemia without apparent focus | 15/113 (13%)  26/113 (23%)  6/113 (5%)  18/113 (16%)  9/113 (8%) 50/113 (44%) | 9/91 (10%)  19/91 (21%)  5/91 (6%)  16/91 (18%)  7/91 (8%) 43/91 (47%) | 6/22 (27%)  7/22 (32%)  1/22 (5%)  2/22 (9%)  2/22 (9%)  7/22 (32%) | **0.031**  0.274  1.000  0.518  1.000  0.191 |
| **Disease severity**  - Intensive care | 60/110 (55%) | 54/88 (61%) | 6/22 (27%) | **0.004** |
| - Assisted ventilation | 25/106 (24%) | 24/84 (29%) | 1/22 (5%) | **0.022** |
| - Sequelae^1^ 6 months  - Sequelae^1^  12 months | 18/80 (23%)  10/76 (13%) | 16/63 (25%)  9/59 (15%) | 2/17 (12%)  1/17 (6%) | 0.333  0.441 |
| - Mortality^2^ <30 days  - Mortality^2^ <24 hours | 16/113 (14%)  9/113 (8%) | 15/91 (17%)  9/91 (10%) | 1/22 (5%)  0/22 (0%) | 0.190  0.202 |

Differences between groups analysed using the Chi^2^ test or Fisher’s exact test. For comparison of non-parametric median values, the Mann–Whitney U test was used. A *p-*value <0.05 was considered statistically significant, presented in bold.

^1^ Active smoker or passively exposed to cigarette smoke.

^2^ Due to diabetes (n=8), malignancy (n=3), splenectomized state (n=2), immunosuppressive treatment (immunomodulating drugs, cytostatic drugs or corticosteroids equivalent to ≥10 mg prednisolone daily) (n=2) or alcohol abuse (n=1). None had verified complement deficiency.

^3^ Results of first test at hospital.

^4^ The assessments of the sites of infection were based on the retrospective review of the medical records. Except for bacteraemia without apparent focus, more than one site of infection is possible.

Supplementary Table S3. Characteristics of patients with meningococcal serogroup W cc11 infections (n=91), 2013 strain compared to other cc11 isolates.

|  | **All cc11**  **(n=91)** | **2013 strain**  **(n=68)** | **Cc11 non-2013 strain**  **(n=23)** | ***p-*value** |
| --- | --- | --- | --- | --- |
| **Baseline characteristics**  - Age: median (25th-75^th^ percentile), range, years | 46 (19-71),  0-95 | 52 (20-73),  12-95 | 22 (4-46),  0-79 | **0.001** |
| - Female gender | 51/91 (56%) | 40/68 (59%) | 11/23 (48%) | 0.358 |
| - Living alone | 22/83 (27%) | 15/60 (25%) | 7/23 (30%) | 0.616 |
| - Smoker^1^ | 4/64 (6%) | 4/48 (8%) | 0/16 (0%) | 0.564 |
| - Immunocompromised state^2^ | 11/91 (12%) | 11/68 (16%) | 0/23 (0%) | 0.059 |
| **Symptoms and clinical findings**  - Diarrhoea  - Vomiting  - Diarrhoea and/or vomiting  - Headache  - Sore throat  - Cough  - Decreased level of consciousness  - Petechiae | 19/88 (22%)  31/88 (35%)  39/88 (44%)  22/88 (23%)  34/88 (39%)  22/88 (25%)  22/86 (26%)  2/80 (3%) | 16/65 (25%)  24/65 (37%)  29/65 (45%)  17/65 (26%)  28/65 (43%)  18/65 (28%)  15/65 (23%)  2/60 (3%) | 3/23 (13%)  7/23 (30%)  10/23 (44%)  3/23 (13%)  6/23 (26%)  4/23 (17%)  7/21 (33%)  0/20 (0%) | 0.378  0.576  0.925  0.255  0.150  0.409  0.349  1.000 |
| **Laboratory results**^3^  - Platelet count <150 x 10^9^/L  - Lactate >4 mmol/L | 42/87 (48%)  34/67 (51%) | 33/64 (52%)  28/53 (53%) | 9/23 (39%)  6/14 (43%) | 0.306  0.507 |
| **Time from onset of symptoms to arrival at hospital**  - Median (25-75^th^ percentile), days | 1 (0-2) | 1 (0-2) | 1 (0-3) | 0.364 |
| **Time from arrival at hospital to antibiotic treatment**  - Median (25^th^-75^th^ percentile), hours | 1 (1-4) | 1 (1-3) | 2 (1-4) | 0.372 |
| **Sites of infection^4^**  - Meningitis  - Pneumonia  - Arthritis  - Throat infection  - Epiglottitis  - Bacteraemia without apparent focus | 9/91 (10%)  19/91 (21%)  5/91 (6%)  16/91 (18%)  7/91 (8%)  43/91 (47%) | 5/68 (7%)  15/68 (22%)  3/68 (4%)  13/68 (19%)  6/68 (9%)  33/68 (49%) | 4/23 (17%)  4/23 (17%)  2/23 (9%)  3/23 (13%)  1/23 (4%)  10/23 (44%) | 0.223  0.772  0.597  0.752  0.674  0.675 |

Differences between groups analysed using the Chi^2^ test or Fisher’s exact test. For comparison of non-parametric median values, the Mann–Whitney U test was used. A *p-*value <0.05 was considered statistically significant and presented in bold.

^1^ Active smoker or passively exposed to cigarette smoke.

^2^ Due to diabetes (n=5), malignancy (n=2), splenectomized state (n=2), immunosuppressive treatment (immunomodulating drugs, cytostatic drugs or corticosteroids equivalent to ≥10 mg prednisolone daily) (n=1) or alcohol abuse (n=1). None had verified complement deficiency.

^3^ Results of first test at hospital.

^4^ The assessments of the sites of infection were based on the retrospective review of the medical records. Except for bacteraemia without apparent focus, more than one site of infection is possible.

Supplementary Table S4. Baseline characteristics of all 134 patients with MenW in Sweden 1995–2019.

|  | **Total**  **(n=134)** | **Missing (n=21)** | ***p*-value** | **Cc-11**  **(n=99)** | **Non cc-11 (n=35)** | ***p-*value** | **2013 strain (n=72)** | **Non-2013 strain (n=62)** | ***p*-value** |
| --- | --- | --- | --- | --- | --- | --- | --- | --- | --- |
| **Age, years**  - Median,  (25^th^-75^th^ percentile),  range | 48  (19-73)  0–95 | 48  (19-75)  1-93 | 0.833 | 44  (19-70)  0–95 | 63  (16-79)  0–94 | 0.225 | 52  (20-73)  12–95 | 41  (4-74)  0–94 | 0.093 |
| **Female gender** | 77/134 (57%) | 12/21  (77%) | 1.000 | 60/99 (61%) | 17/35 (49%) | 0.216 | 44/72 (61%) | 33/62  (53%) | 0.357 |
| **Mortality** | 20/134 (15%) | 4/21  (19%) | 0.519 | 18/99 (18%) | 2/35  (6%) | 0.099 | 12/72 (17%) | 8/62  (13%) | 0.542 |

Data analysed using the Chi^2^ test or Fisher’s exact test. For comparisons of median values, Mann Whitney U test was used; a *p*-value <0.05 was considered significant. Cc = clonal complex.

Supplementary Table S5. Disease severity of *Neisseria meningitidis* serogroup W infections in relation to site of infection

|  | |  | **Total MenW (n=113)** | | | | |
| --- | --- | --- | --- | --- | --- | --- | --- |
|  |  | | **Bacteraemia without apparent focus (n=50)** | **Meningitis^1^**  **(n=15)** | **Pneumonia^2^**  **(n=26)** | **Epiglottitis^2^**  **(n=9)** | **Arthritis (n=6)^3^** |
| **Mortality**  **<30 days**  **<24 h** | 16/113 (14%)  9/113 (8%) | | 13/50 (26%) ***p=*0.001**  9/50 (18%) ***p=*0.000** | 1/15 (7%) *p=*0.691  0/15 (0%) *p=*0.605 | 1/26 (4%) *p=*0.113  0/26 (0%) *p=*0.115 | 1 (11%) *p=*1.000  0 (0%)  *p=*1.000 | 0/6 (0%) *p=*0.592  0/6 (0%) *p=*1.000 |
| **Intensive care** | 60/110 (55%) | | 27/48 (56%) *p=*0.752 | 11/14 (78%) *p=*0.053 | 12/26 (46%) *p=*0.325 | 7/9 (78%) *p=*0.178 | 3/6 (50%) *p=*1.000 |
| **Assisted ventilation** | 25/106 (24%) | | 17/45 (38%) ***p=*0.003** | 2/14 (14%) *p=*0.511 | 2/26 (8%)  ***p=*0.033** | 4/9 (44%) *p=*0.210 | 0/6 (0%) *p=*0.332 |
|  |  | | **2013 strain (n=68)** | | | | |
|  |  | | **Bacteraemia without apparent focus (n=33)** | **Meningitis^1^**  **(n=5)** | **Pneumonia^2^**  **(n=15)** | **Epiglottitis^2^**  **(n=6)** | **Arthritis (n=3)^3^** |
| **Mortality**  **<30 days**  **<24 h** | 13/68 (19%)  8/68 (12%) | | 12/33 (36%) ***p*=0.000**  8/33 (24%) ***p*=0.002** | 0/5 (0%) *p*=0.575  0/5 (0%) *p*=1.000 | 1/15 (7%) *p*=0.269  0/15 (0%) *p*=0.184 | 0/6 (0%) *p*=0.587  0/6 (0%) *p*=1.000 | 0/3 (0%) *p*=1.000  0/3 (0%) *p*=1.000 |
| **Intensive care** | 41/66 (62%) | | 20/31 (65%) *p*=0.706 | 4/5 (80%) *p*=0.642 | 9/15 (60%) *p*=0.847 | 5/6 (83%) *p*=0.396 | 2/3 (67%) *p*=1.000 |
| **Assisted ventilation** | 19/60 (30%) | | 14/29 (48%) ***p*=0.004** | 1/5 (20%) *p*=1.000 | 1/15 (7%) ***p*=0.026** | 3/6 (50%) *p*=0.355 | 0/3 (0%) *p*=0.547 |

The group of patients with the given site of infection respectively have been compared with the patients without the site of infection. The Chi^2^ test, or Fisher’s exact test if sample size was small, was used for comparison between groups; *p*<0.05 was considered statistically significant and presented in bold.

^1^ Meningitis was assessed if MenW was detected in cerebrospinal fluid or clinical picture of meningitis in combination with MenW bacteraemia.

^2^ Pneumonia and epiglottitis were both combined with bacteraemia.

^3^ Arthritis was assessed if MenW was detected in joint fluid by culture or PCR.

Supplementary Table S6. Mortality within 30 days and 1 day of admission respectively for patients with meningococcal serogroup W infections in Sweden 1995–2019, in relation to patient factors and observations of clinical signs and biomarkers, first test at hospital.

|  | **Mortality <30 d**  All patients 16/113 (14%) | **Mortality <1 d**  All patients 9/113 (8%) |
| --- | --- | --- |
| **Temperature** >38 vs temp ≤38  <37 vs temp ≥37 | 7/71 (10%) *p=*0.086  3/12 (25%) *p=*0.208 | 4/71 (6%) *p=*0.241  1/12 (8%) *p=*1.000 |
| **WBC count**   - <4 - 4–12 - >12 | 9/20 (45%) ***p=*0.000**  5/34 (15%) *p=*0.868  1/54 (2%) ***p=*0.000** | 7/20 (35%) ***p=*0.000**  1/34 (3%) *p=*0.431  0/54 (0%) ***p=*0.000** |
| **Platelet count**   - <20 - <50 - <100 - <150 | 4/9 (44%) ***p=*0.023**  7/20 (35%) ***p=*0.003**  10/29 (35%) ***p=*0.000**  13/44 (30%) ***p=*0.000** | 2/9 (22%) *p=*0.140  4/20 (20%) ***p=*0.041**  7/29 (24%) ***p=*0.000**  8/44 (18%) ***p=*0.001** |
| **Lactate**   - >2 - >3 - >4 | 14/51 (28%) ***p=*0.028**  13/41 (32%) ***p=*0.008**  13/34 (38%) ***p=*0.000** | 8/51 (16%) *p=*0.052  8/41 (20%) ***p=*0.007**  8/34 (24%) ***p=*0.001** |
| **Age**   - 0–14 - 15–24 - 25–59 - 60– | 1/15 (7%) *p=*0.691  2/28 (7%) *p=*0.350  5/24 (25%) *p=*0.086  7/46 (15%) *p=*0.789 | 0/15 (0%) *p=*0.605  1/28 (4%) *p=*0.447  5/24 (21%) ***p=*0.020**  3/46 (7%) *p=*0.736 |
| **Immunocompromised state^1^** | 2/16 (13%) *p=*1.000 | 1/16 (6%) *p=*1.000 |
| **Decreased level of consciousness** | 9/25 (36%) ***p=*0.000** | 5/25 (20%) ***p=*0.016** |
| **Respiratory rate** >22 / min | 9/49 (18%) *p=*0.343 | 6/49 (12%) *p=*0.077 |

The Chi2 test, or Fisher’s exact test if sample size was small, was used for comparison between groups; p<0.05 was considered statistically significant and is presented in bold.

^1^ Due to diabetes (n=1) or malignancy (n=1).
